# Exploratory Evaluation of Circulating Microbiota-Derived Corisin Levels in Women with Adverse Pregnancy Outcomes

**DOI:** 10.3390/antiox14060670

**Published:** 2025-05-31

**Authors:** Maya Kato, Masafumi Nii, Kuniaki Toriyabe, Yuya Tamaishi, Sho Takakura, Shoichi Magawa, Taro Yasuma, Corina N. D’Alessandro-Gabazza, Hajime Fujimoto, Masaaki Toda, Isaac Cann, Tetsu Kobayashi, Esteban C. Gabazza, Eiji Kondo, Tomoaki Ikeda

**Affiliations:** 1 Department of Obstetrics and Gynecology, Faculty and Graduate School of Medicine, Mie University, Mie University Hospital, Mie 514-8507, Japan; 2 Department of Immunology, Faculty and Graduate School of Medicine, Mie University, Edobashi 2-174, Tsu, Mie 514-8507, Japan; 3 Department Pulmonary and Critical Care Medicine, Faculty and Graduate School of Medicine, Mie University, Edobashi 2-174, Tsu, Mie 514-8507, Japan; 4 Microbiome Research Center, Mie University, Edobashi 2-174, Tsu, Mie 514-8507, Japan; 5 Department of Diabetes, Endocrinology and Metabolism, Faculty and Graduate School of Medicine, Mie University, Edobashi 2-174, Tsu, Mie 514-8507, Japan; 6 Department of Microbiology, University of Illinois at Urbana-Champaign, Urbana, IL 61820, USA

**Keywords:** corisin, preterm birth, low birth weight infants, small for gestational age, microbiota, pregnancy

## Abstract

Preterm birth and low birth weight remain major contributors to neonatal morbidity and mortality, yet the underlying mechanisms are not fully understood. Maternal microbiota has been implicated in adverse pregnancy outcomes, but key mediators remain unidentified. We previously showed that the microbiota-derived peptide corisin induces epithelial apoptosis via mitochondrial membrane depolarization and reactive oxygen species accumulation. In this retrospective preliminary study, we evaluated the association between maternal serum corisin levels and pregnancy outcomes in 84 eligible women. Among them, 10 experienced preterm birth, and 22 delivered low-birth-weight infants. Corisin levels were significantly elevated in these groups compared with women with full-term, normal-weight deliveries. Preterm birth was associated with increased tissue factor, while low birth weight correlated with higher thrombin–antithrombin complex and soluble thrombomodulin and lower fibrinogen levels. Corisin concentrations showed negative correlations with maternal BMI, birth weight and length, and estimated fetal weight. Positive correlations were observed between corisin, myeloperoxidase, and several coagulation markers. These preliminary findings suggest that elevated maternal corisin levels are associated with adverse pregnancy outcomes and may reflect underlying mechanisms involving oxidative stress and coagulation activation. Further investigation is warranted to clarify its potential role as a microbiota-derived biomarker in pregnancy complications.

## 1. Introduction

Neonates with low birth weight (LBW) represent a significant global health concern due to their increased risk of perinatal mortality and long-term health complications [1]. Preterm birth and being small for gestational age are major contributors to low birth weight, which remains a leading cause of mortality in children under five years of age [2,3]. These infants are at a heightened risk for a spectrum of health issues later in life, including developmental delays and gastrointestinal, respiratory, cardiovascular, behavioral, and metabolic disorders [4,5,6,7,8]. The burden of LBW and preterm birth varies significantly by income level: over 90% of preterm births and LBW infants occur in low- and middle-income countries, particularly in South Asia and sub-Saharan Africa, while high-income countries tend to report lower prevalence due to better access to antenatal care and neonatal services [9,10]. Current estimates indicate that annually, there are more than 13 million preterm births and more than 23 million infants born with size small worldwide, underscoring the urgent need for enhanced prenatal care and postnatal interventions to improve outcomes for these vulnerable populations [11,12,13]. Longitudinal studies suggest that these early life challenges can have lasting effects, potentially influencing educational achievements, economic productivity, and overall quality of life [1,14,15]. Hence, it is imperative to conduct thorough research to unravel the complex pathophysiological consequences and the root causes associated with preterm and LBW.

The etiology of preterm birth and LBW is multifactorial, involving a complex interplay of genetic, environmental, and maternal health factors. Preterm delivery may occur spontaneously or as a consequence of maternal conditions such as diabetes mellitus, hypertension, or infections [16,17]. Similarly, LBW can result from chromosomal abnormalities or maternal complications, including malnutrition, anemia, and infection [18,19]. Additional contributors include excessive oxidative stress, placental insufficiency, pregnancy complications (e.g., preeclampsia), and lifestyle factors (e.g., smoking), all of which significantly increase the risk of adverse perinatal outcomes [20,21,22,23,24]. Increasing attention has also been drawn to the role of the maternal microbiome, particularly gut microbiota dysbiosis, in the pathogenesis of these complications [25,26,27,28,29]. Disruptions in the maternal microbiota are now recognized as a critical source of oxidative stress, a key pathological mechanism implicated in impaired fetal development. Emerging evidence has demonstrated that gut microbiota-mediated oxidative damage plays a pivotal role in various pregnancy-related disorders [30,31,32,33,34,35]. For instance, bisphenol A-induced oxidative stress has been linked to fetal growth restriction via alterations in gut microbiota [33]. Maternal obesity, frequently associated with dysbiosis, has been shown to enhance placental oxidative stress, thereby exacerbating the risk of poor fetal outcomes [32]. Furthermore, interventions such as malic acid supplementation have been reported to mitigate oxidative stress through microbiota modulation, while gestational diabetes has been associated with a pathological triad of microbiota imbalance, inflammation, and oxidative injury [30,31,33].

In addition, our recent research has identified a microbiota-derived peptide, corisin, which induces apoptosis through oxidative stress and mitochondrial membrane depolarization in parenchymal cells across multiple organs, including the lungs, kidneys, intestines, eyes, and skin [36,37]. Mechanistically, corisin disrupts mitochondrial integrity by decreasing mitochondrial membrane potential, leading to the release of cytochrome c and activation of caspase-dependent apoptotic pathways. It also promotes the generation of reactive oxygen species (ROS), upregulates pro-apoptotic proteins such as BAX, and downregulates anti-apoptotic BCL-2 family members, thereby tipping the balance toward cell death [34,35]. Initially discovered as a fragment of bacterial transglycosylases [38], corisin was first attributed to *Staphylococcus nepalensis* strain CDNG. However, subsequent studies have demonstrated that a broader range of commensal, opportunistic, and pathogenic bacteria, such as *Staphylococcus haemolyticus*, *Staphylococcus cohnii*, and *Mycobacterium abscessus*, can also produce this peptide [36,38]. Beyond its cytotoxic effects, corisin exhibits potent pro-inflammatory, pro-fibrotic, and procoagulant properties, promoting the expression of pro-inflammatory cytokines, growth factors, and tissue factor [36,38,39]. Its pathogenic role has also been confirmed in experimental models of acute lung injury and acute exacerbation of pulmonary fibrosis [38,39,40]. The pathogenic role of corisin has also been substantiated in experimental models of acute lung injury and acute exacerbation of pulmonary fibrosis [36,38,41].

Previous studies suggest that intestinal barrier dysfunction, particularly increased permeability associated with dysbiosis-induced leaky gut, may facilitate the systemic translocation of corisin from the gut lumen into the bloodstream [39]. For example, we previously found that circulating levels of corisin and fatty acid binding protein 2 (FABP2), an established marker of intestinal permeability, were both elevated and strongly correlated in COVID-19 patients and in mice with SARS-CoV-2 spike protein-induced organ injury, supporting gut barrier leakage as a contributor to systemic corisin levels. Notably, pregnancy is characterized by profound shifts in gut microbial composition and intestinal barrier integrity, with dysbiosis-related permeability increasingly implicated in adverse pregnancy outcomes [42,43]. Building upon this background, we hypothesized that circulating levels of corisin may be associated with abnormal pregnancy outcomes. This preliminary retrospective study, therefore, aims to investigate the potential link between systemic corisin levels and pregnancy-related complications.

## 2. Materials and Methods

### 2.1. Study Population

This pilot retrospective study enrolled 105 women who consulted or were referred to our institution from December 2023 through July 2024 (Figure S1). Inclusion criteria involved women who had delivered live infants and the availability of blood samples. Blood sampling was conducted within 4 h postpartum. Pregnant women without sufficient remaining blood for corisin measurement and those with twin pregnancies, cervical cerclage, or suspected bacteremia were excluded from the study. Clinical, demographic, and laboratory data were retrieved from electronic medical records, including underlying diseases, pregnancy complications, and obstetric and perinatal information. Gestational age was determined based on the last menstrual period. The primary outcomes of this study were preterm and low birth weight. Preterm birth is defined as delivery occurring before the completion of 37 weeks of gestation, whereas full-term birth is defined as delivery occurring between 37 and 41 completed weeks of gestation [44,45]. LBW refers to a birth weight of less than 2500 g, irrespective of gestational age [44,45]. Small for gestational age is characterized by a birth weight below the 10th percentile for gestational age and sex [46]. Pre-pregnancy body mass index (BMI) was calculated by dividing body weight (kg) by the square of height (m^2^).

### 2.2. Ethics Approval and Consent to Participate

This study was approved by the Mie University Ethical Committee for Clinical Investigation (approval number: H2024-137; date of approval: 19 August 2024) and was conducted in accordance with the principles outlined in the Declaration of Helsinki. The requirement for informed consent was waived by the committee (Mie University Ethical Committee for Clinical Investigation) due to the retrospective design of the study.

### 2.3. Laboratory Analysis

Blood samples collected from the patients were sent to the Clinical Laboratory of the University Hospital. A portion of each sample was aliquoted for routine hemograms and biochemical analyses, while the remaining portion was specifically allocated and promptly stored at −80 °C for designated future analyses. The levels of tissue factor (TF), soluble thrombomodulin (TM), and fatty acid binding protein-2, also named intestinal-type fatty acid binding protein (I-FABP), were measured using enzyme immunoassay kits from R&D Systems (Minneapolis, MN, USA); thrombin–antithrombin complex (TAT) was quantified using a kit from Affinity Biologicals (Ancaster, ON, Canada); and myeloperoxidase (MPO) was measured using an ELISA kit from Immunodiagnostic Ag (Bensheim, Germany). The levels of corisin were measured in the serum sample using an in-house enzyme immunoassay that we previously developed and reported [36]. Briefly, a 96-well plate was coated with a polyclonal anti-transglycosylase antibody (2.5 µg/mL) in phosphate-buffered saline (PBS) and incubated at 4 °C for 18 h. After five PBS washes, a blocking buffer was added, followed by 2 h of incubation at room temperature. Diluted samples or standard synthetic corisin were then applied, and the plate was incubated at 4 °C for 18 h. After four PBS washes, a biotin-labeled anticorisin monoclonal antibody (2.5 µg/mL) was added and incubated for 2 h at room temperature. Streptavidin-horse radish peroxidase was applied for 1 h, followed by color development. Absorbance at 450 nm was measured using a BIOD-RAD iMarkTM microplate reader (Bio-Rad Laboratories, Inc.; Hercules, CA, USA). Corisin concentrations were calculated from a standard curve, with intra- and inter-assay CVs under 10%.

### 2.4. Statistical Analysis 

Data are presented as the mean ± standard error of the mean (SEM) or as the median with interquartile ranges for continuous variables and as percentages for categorical variables. Normality was assessed using the Kolmogorov–Smirnov and Shapiro–Wilk tests. For comparisons between two groups, either an unpaired *t*-test or the Mann–Whitney U test was used, depending on the distribution of the data. Differences among three or more groups were analyzed using one-way analysis of variance (ANOVA), followed by Tukey’s post hoc test. The Spearman correlation or the Pearson product–moment correlation of log-transformed data was used to evaluate the strength of relationships between variables. The significant differences in the frequencies of categorical variables were assessed using Fisher’s exact test. We conducted an exploratory assessment of variables associated with birth weight using multiple linear regression analysis. Several models were constructed to examine whether serum corisin was independently associated with birth weight when considered alongside other statistically significant variables identified in univariate analyses. All statistical analyses were performed using GraphPad Prism version 10.4.1 (San Diego, CA, USA). A *p*-value of <0.05 was considered statistically significant.

## 3. Results

### 3.1. Study Population Characteristics

Of the initial cohort of 105 subjects, 84 (34.7 ± 4.8 years old) met the eligibility criteria for inclusion in the study analyzing microbiota-derived corisin (Figure S1). Within this group, 10 experienced preterm births, while 74 had full-term deliveries (Table 1). Additionally, 22 participants delivered low-birth-weight infants, whereas 62 delivered newborns weighing more than 2.5 kg (Table 2). Among the small-for-gestational-age infants, seven had a birth weight below 2.5 kg, and one had a birth weight above 2.5 kg. A total of 18 subjects were diagnosed with chorioamnionitis, with or without accompanying chorangiosis.

### 3.2. Comparative Analysis of Clinical Data in Full-Term and Preterm Births

Prenatal and perinatal data revealed no significant statistical differences in the prevalence of underlying health conditions, complications, number of small-for-gestational-age infants, or methods of delivery and conception between the full-term and preterm birth groups (Table 1). However, systolic blood pressure and blood glucose levels were significantly higher, while serum potassium levels were markedly lower in the preterm birth cohort compared with the full-term group (Table S1). Additionally, placental weight, estimated fetal weight assessed via ultrasonography, and neonatal birth weight, length, head circumference, and Apgar scores were significantly reduced in preterm births compared with full-term births (Table S1).

### 3.3. Comparative Analysis of Clinical Data in Patients Delivering Neonates Weighing More or Less than 2.5 kg

The analysis of clinical data revealed no statistically significant differences in the frequency of underlying medical conditions or the methods of delivery and conception between patients delivering neonates weighing less than 2.5 kg and those delivering neonates weighing more than 2.5 kg. However, patients who delivered low-birth-weight infants experienced significantly more complications and had a significantly higher number of small-for-gestational-age infants compared with those who delivered infants weighing more than 2.5 kg (Table 2). Furthermore, BMI and platelet counts were significantly lower, while activated partial thromboplastin time was markedly prolonged and prothrombin time significantly shortened in patients delivering neonates weighing less than 2.5 kg compared with those delivering neonates weighing more than 2.5 kg (Table S2). Additionally, estimated fetal weight, placental weight, birth weight, birth height, head circumference, and Apgar scores were significantly lower in patients delivering neonates weighing less than 2.5 kg than those delivering neonates weighing more than 2.5 kg (Table S2).

### 3.4. Elevated Circulating Corisin Levels in Patients with Adverse Pregnancy Outcomes

**Figure 1:**
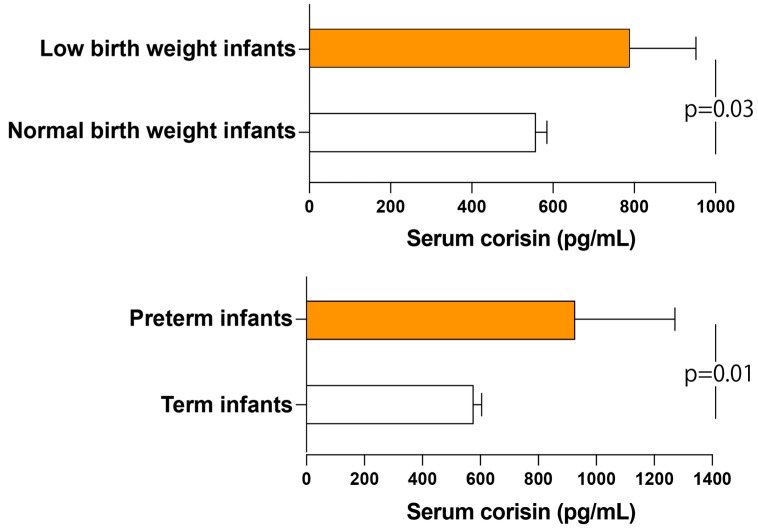
Increased serum levels of corisin in women with adverse pregnancy outcomes. Corisin levels were measured using enzyme immunoassays as described in the Materials and Methods section. Measurements were conducted in patients delivering infants with a birth weight of less than 2.5 kg (*n* = 22) or greater than 2.5 kg (*n* = 62) and preterm (*n* = 10) or term (*n* = 74) newborns. Data are presented as mean ± SEM. Statistical analysis was performed using an unpaired *t*-test.

Serum corisin levels were significantly elevated in individuals who experienced preterm birth or delivered low-birth-weight infants compared with those with full-term deliveries and neonates weighing more than 2.5 kg (Figure 1). Conversely, serum corisin concentrations did not exhibit statistically significant variations across subjects stratified by the presence or absence of pregnancy-related complications or comorbidities, nor among individuals presenting with both conditions. Similarly, no significant differences in serum corisin levels were observed between subjects diagnosed with chorioamnionitis and/or chorangiosis and those without these conditions (Figure S2).

### 3.5. Correlation Between Serum Myeloperoxidase and Corisin Levels

**Figure 2:**
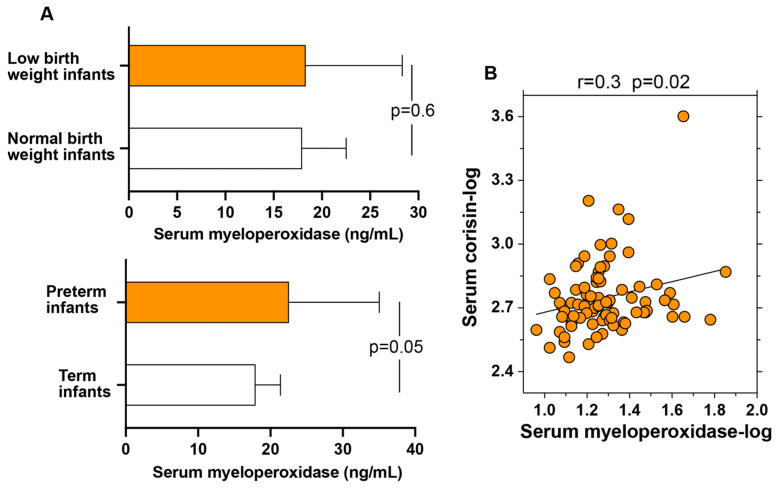
Elevated serum levels of myeloperoxidase in women delivering preterm infants and significant correlation between the serum levels of myeloperoxidase and corisin in all patients. (**A**,**B**) Myeloperoxidase and corisin levels were measured using enzyme immunoassays as described in the Materials and Methods section. Measurements were conducted in patients delivering infants with a birth weight of less than 2.5 kg (*n* = 22) or greater than 2.5 kg (*n* = 62) pre-term (*n* = 10) or term (*n* = 74) newborns. Data are presented as median with the interquartile range. Statistical analysis was performed using the Mann–Whitney U test.

Serum levels of myeloperoxidase, an established biomarker of oxidative stress, showed a strong tendency to be elevated in pregnant women who delivered preterm infants compared with those who delivered at term, with the difference approaching statistical significance (*p* = 0.05). In contrast, no significant difference in serum myeloperoxidase levels was observed between women who delivered neonates weighing less than 2.5 kg and those with neonates weighing more than 2.5 kg (Figure 2A). Importantly, serum concentrations of myeloperoxidase were significantly and positively correlated with serum levels of corisin (*p* = 0.02) (Figure 2B). This correlation reinforces the hypothesis that elevated corisin levels may contribute to systemic oxidative stress during pregnancy, particularly in cases complicated by preterm delivery.

### 3.6. Significant Correlation of Serum Corisin Levels with Pregnancy Outcomes

**Figure 3:**
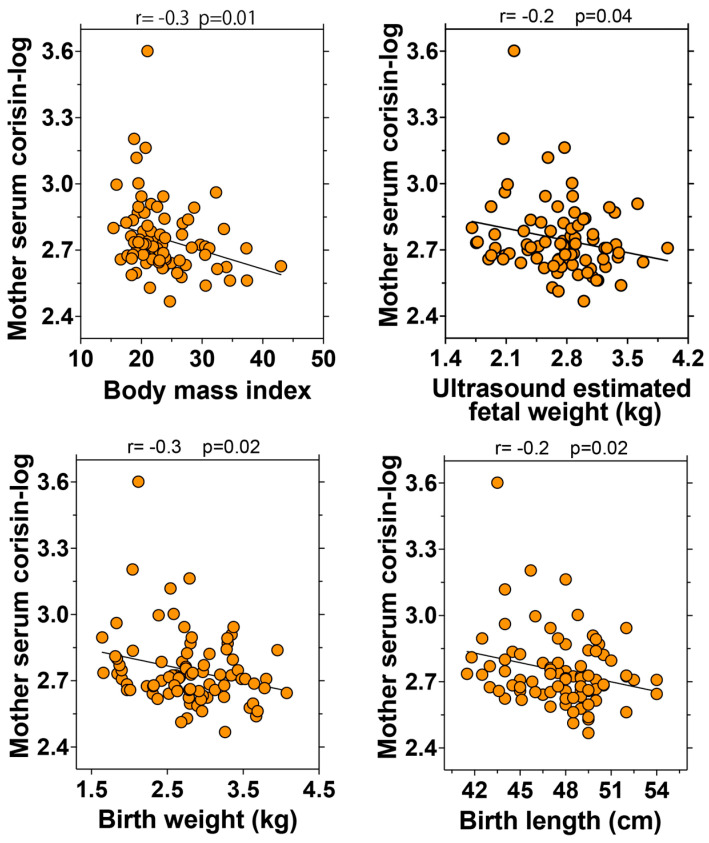
Correlation between corisin and pregnancy outcomes. The relationship between corisin and pregnancy outcomes was evaluated after the log transformation of corisin.

The potential clinical relevance of corisin was assessed by analyzing its relationship with prenatal and perinatal data across all patients. Serum corisin levels demonstrated a significant inverse correlation with the ultrasound-estimated fetal weight (*p* = 0.04), birth weight (*p* = 0.02), and birth height (*p* = 0.02) (Figure 3). Additionally, serum corisin levels showed an inverse correlation with gestational age and the 1 min Apgar score, although these associations did not reach statistical significance (Figure S3). Notably, serum corisin levels were also significantly and inversely correlated with maternal BMI (*p* = 0.01), and this relationship remained robust even after excluding individuals with a BMI greater than 35.

### 3.7. Serum Levels of FABP2, a Marker of Gut Epithelial Permeability, and Corisin

To investigate whether elevated serum corisin levels may be associated with dysbiosis-related increases in intestinal permeability, we measured serum concentrations of intestinal fatty acid-binding protein, also known as fatty acid-binding protein 2 (FABP2), a recognized marker of intestinal epithelial integrity [47]. We then assessed the relationship between FABP2 levels, pregnancy outcomes, and serum corisin concentrations. No significant differences in FABP2 levels were observed between women who delivered preterm versus term infants, nor between those who delivered LBW versus normal birth weight infants (Figure S4A). Furthermore, no correlation was found between serum levels of FABP2 and corisin across the entire cohort (Figure S4B). These results suggest that the elevation in corisin levels observed during pregnancy is unlikely to be attributable to increased intestinal permeability, indicating that alternative mechanisms may underlie corisin accumulation in the maternal circulation.

### 3.8. Coagulation Abnormalities and Their Association with Adverse Pregnancy Outcomes

**Figure 4:**
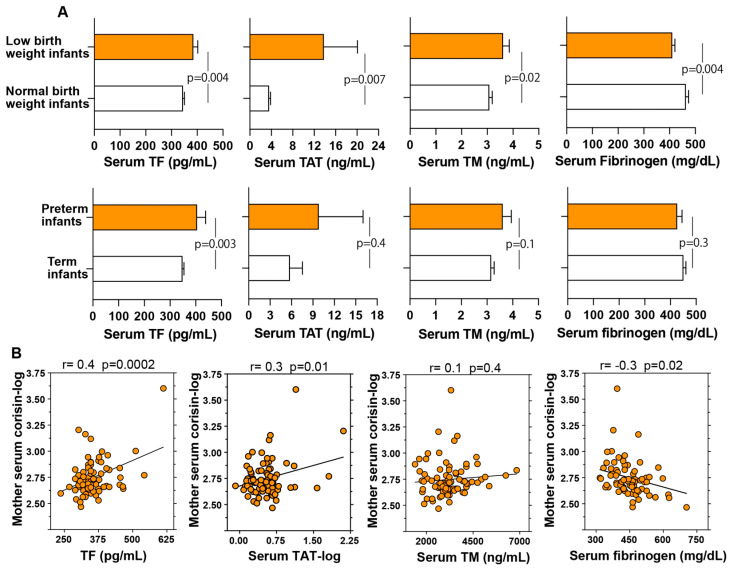
Increased serum levels of coagulation markers in women with adverse pregnancy outcomes. (**A**,**B**) Coagulation marker levels were measured using enzyme immunoassays as described in the Materials and Methods section. Measurements were conducted in patients delivering infants with a birth weight of less than 2.5 kg (*n* = 22) or greater than 2.5 kg (*n* = 62) and preterm (*n* = 10) or term (*n* = 74) newborns. Data are presented as mean ± standard error of the mean, and statistical analysis was performed using Student’s *t*-test.

Alterations in the coagulation system have been implicated in adverse pregnancy outcomes, and corisin has been shown to stimulate the expression of tissue factor, a key initiator of the coagulation cascade [48,49]. In this study, we evaluated serum levels of tissue factor, thrombin–antithrombin complex as a marker of coagulation system activation, and soluble thrombomodulin as a marker of endothelial cell injury in relation to pregnancy outcomes. Fibrinogen, which is typically reduced during coagulation activation due to its conversion to fibrin, was also measured. Serum concentrations of tissue factor, thrombin–antithrombin complex, and soluble thrombomodulin were significantly elevated, whereas fibrinogen levels were significantly decreased in women who delivered low birth weight infants compared with those who delivered infants with normal birth weight. Additionally, tissue factor levels were significantly higher in women with preterm delivery than in those with term delivery (Figure 4A). Although serum levels of thrombin–antithrombin complex, soluble thrombomodulin, and fibrinogen were also elevated in women with preterm delivery, these differences did not reach statistical significance. In addition, serum concentrations of corisin were significantly correlated with serum levels of tissue factor (*p* = 0.0002), thrombin–antithrombin complex (*p* = 0.01), and fibrinogen (*p* = 0.02) (Figure 4B).

### 3.9. Exploratory Multiple Linear Regression Analysis of Factors Associated with Birth Weight

To assess the potential independent association between corisin and birth weight, we performed multiple linear regression analyses using birth weight as a continuous dependent variable. Given the limited number of LBW cases (*n* = 22), we followed the widely accepted guideline of including no more than one independent variable per 10 outcome events to minimize overfitting and ensure model stability [47,48]. Each regression model was, therefore, limited to two independent variables. Candidate variables were selected based on their statistical significance in simple linear regression analyses (*p* < 0.05). To test the robustness of the association, we constructed several models, each including corisin and one additional variable. This approach allowed us to evaluate whether corisin consistently showed an independent association with birth weight while reducing the risk of model overfitting [47].

In the simple linear regression analysis, birth weight was significantly correlated with serum levels of corisin (*p* = 0.02), platelet count (*p* = 0.04), tissue factor (*p* = 0.03), thrombin–antithrombin complex (*p* = 0.04), thrombomodulin (*p* = 0.005), and fibrinogen (*p* = 0.01). In multiple linear regression models limited to two covariates, serum corisin levels remained significantly associated with birth weight after adjusting for platelets, thrombin–antithrombin complex, or thrombomodulin (Table S3). These results indicate a potential association of elevated serum corisin levels with lower birth weight independently from other factors. However, due to the limited number of low birth weight (LBW) cases and the timing of blood sampling, conducted only after delivery, these findings do not establish a definitive predictive value or causal relationship. To validate the clinical utility of serum corisin as a biomarker for adverse pregnancy outcomes, future prospective studies incorporating larger cohorts and longitudinal sampling throughout gestation are essential. Such studies may also help elucidate the temporal dynamics and mechanistic role of corisin in the pathophysiology of fetal growth restriction.

## 4. Discussion

In the present study, serum corisin levels were significantly elevated in pregnant women who experienced preterm birth or delivered low-birth-weight infants compared with controls. Corisin levels also showed significant correlations with birth weight, birth length, myeloperoxidase (a marker of oxidative stress), and markers of coagulation activation, suggesting a potential association between elevated corisin and adverse fetal outcomes.

Extensive research has documented significant alterations in the composition of microbiota at various sites within the body, such as the gut, vagina, endometrium, and oral cavity, during the course of pregnancy [25,27,29,50,51]. Despite these findings, the exact impact of these microbial shifts on the pathophysiology of birth outcomes remains a subject of ongoing investigation. Historically, the bulk of research efforts have been directed toward establishing links between pregnancy-related complications and the increase or decrease of particular bacterial populations within the body’s microbiota, assessed at different levels of the bacterial taxonomic hierarchy [52,53,54]. Recently, the scientific community has turned its attention towards the potential roles played by secretory products derived from the microbiota in pregnancy and offspring health [25,55].

Emerging evidence suggests that microbial antigens, peptides, and metabolites, including short-chain fatty acids and antimicrobial peptides, modulate inflammatory responses, fostering immune tolerance and influencing placental functionality [50,56]. Intrauterine inflammation, triggered by bacterial lipoproteins and lipopolysaccharides, and oxidative stress have been implicated in preterm births [21,57,58,59]. Animal models have demonstrated that increased levels of trimethylamine-N-oxide, which induces oxidative stress, along with decreased levels of acetate and butyrate, can contribute to adverse offspring outcomes [60]. Furthermore, vaginal dysbiosis during pregnancy, characterized by reduced *Lactobacillus* populations, decreased lactic acid production, elevated vaginal pH, and abnormal secretion of short-chain fatty acids by anaerobic bacteria, creates a microenvironment conducive to inflammatory mediator secretion, potentially triggering preterm labor [27,61,62,63]. In parallel, the increasing maternal age observed in many populations over recent decades has emerged as an independent risk factor for adverse birth outcomes. Advanced maternal age (typically defined as ≥35 years) has been consistently associated with a higher incidence of preterm birth and LBW, potentially due to age-related changes in vascular function, oxidative stress, and uterine–placental perfusion [64,65,66,67]. Thus, both maternal age and microbiota-derived factors may independently or synergistically modulate the risk of preterm birth and fetal growth restriction.

Given the growing evidence linking microbial dysbiosis to pregnancy complications, identifying a quantifiable microbial product in blood could serve as a valuable biomarker. Such a marker could aid in the early detection of at-risk pregnancies, guide monitoring or interventions, and provide insight into underlying mechanisms. Previous studies have examined microbial components as predictive markers. For example, serum antibodies against lipopolysaccharide (LPS) and cervicovaginal short-chain fatty acids (SCFAs) have been proposed to identify women at risk of adverse outcomes [68,69]. Although elevated SCFAs in vaginal fluid may predict preterm birth, clinical implementation is limited due to invasive sampling and analytical complexity [70]. Recently, integrative approaches combining immune, microbial, and metabolic markers have shown improved predictive value [71], highlighting the potential of circulating dysbiosis-related markers. In this context, we conducted a preliminary study to explore corisin as a candidate biomarker. Corisin is a 2 kDa peptide composed of a conserved 19-amino-acid sequence released via the degradation of bacterial transglycosylases [38]. Elevated levels of corisin and related peptides have been linked to dysbiosis-associated inflammatory conditions, including pulmonary fibrosis, cholangitis, and viral infections [36,37,38,39,40,41]. We hypothesized that serum corisin, as a marker of microbial dysbiosis, may correlate with adverse pregnancy outcomes. Indeed, women with preterm birth or low-birth-weight neonates showed significantly higher serum corisin levels. Corisin concentrations were found to be inversely associated with birth weight, and this association remained significant after adjusting for other variables in multiple linear regression models, suggesting a possible link to impaired fetal development. However, as correlation does not imply causation, these findings should be interpreted with caution. Further prospective studies with larger cohorts are warranted to validate this association and explore the underlying mechanisms.

In our study, we observed a significant inverse correlation between maternal BMI and serum corisin levels during pregnancy. Notably, this relationship remained consistent even after excluding participants with extremely high BMI values, suggesting a robust association across the full BMI spectrum. The inverse correlation suggests a potential link between higher maternal BMI and alterations in the gut microbiota, which may affect corisin production. Increased BMI is known to induce notable changes in gut microbial composition, including reduced diversity and shifts in bacterial taxa, typically involving a decrease in beneficial commensals and an increase in pro-inflammatory species [72,73]. Given that corisin is produced by *Staphylococcus nepalensis*, its lower levels in women with higher BMI may reflect reduced abundance or activity of this bacterial species in the gut. BMI-related dysbiosis can alter the metabolic output of the gut microbiome, affecting levels of bioactive metabolites that influence host physiology [74]. Thus, decreased corisin levels may be a downstream consequence of microbial dysregulation associated with elevated BMI, potentially contributing to changes in the maternal immune environment. Further studies in larger populations are needed to clarify the mechanisms underlying this association.

Corisin was initially identified for its ability to induce apoptosis through mitochondrial oxidative stress in parenchymal cells across multiple organs and has since been implicated in inflammation, tissue injury, and fibrosis [36,37,38,41]. Later studies revealed its role in triggering the coagulation cascade via upregulation of tissue factor, a key initiator of coagulation [39,75]. In the present study, we extend the pathophysiological relevance of corisin by providing, to our knowledge, the first evidence suggesting its potential involvement in adverse pregnancy outcomes. Although the underlying mechanisms remain unclear, its known pro-oxidative, pro-apoptotic, pro-inflammatory, and procoagulant activities make it a plausible contributor. These processes are central to pregnancy-related disorders such as recurrent miscarriage, preeclampsia, and placental insufficiency [48,49,76,77,78,79].

Trophoblast apoptosis, though essential to placental development, may impair placental invasion and spiral artery remodeling when excessive, leading to reduced perfusion, oxidative stress, inflammation, and coagulation activation [76,77,79]. Concurrently, the coagulation cascade may become pathologically hyperactivated in response to inflammatory stimuli and vascular injury [49]. The placenta’s inherently procoagulant state further predisposes to pathological thrombosis, infarction, and fetal growth restriction [49,77]. In line with this, we observed elevated levels of coagulation activation markers in women delivering low birth weight infants, along with significant correlations with serum corisin levels. Nonetheless, given the observational nature of this study, causality cannot be inferred. Future research should clarify corisin’s signaling pathways at the maternal–fetal interface and assess its levels in maternal serum, cervicovaginal fluid, amniotic fluid, and placental tissues throughout gestation.

Although increased gut permeability is a plausible route for microbiota-derived peptides to enter circulation, our findings do not support this mechanism. Serum levels of fatty acid-binding protein 2 (FABP2), a validated marker of intestinal epithelial integrity [47], showed no significant differences between women with adverse pregnancy outcomes and controls, nor did they correlate with serum corisin levels. This suggests that intestinal barrier dysfunction is unlikely to explain the observed increase in circulating corisin. Alternative explanations include increased production or release from corisin-producing bacteria residing in extraintestinal sites such as the skin, respiratory tract, or reproductive tract, which are known to harbor staphylococcal species and undergo microbial shifts during pregnancy or inflammation [80,81,82]. Additionally, altered host–microbial interactions, immune modulation, or impaired hepatic or renal clearance may contribute to elevated serum levels. These hypotheses warrant further investigation in mechanistic and prospective studies.

The major strengths of the current study include its innovative approach of targeting a microbiome-derived factor measurable in peripheral blood and its potential for clinical applicability. The ability to measure corisin levels using a well-characterized enzyme immunoassay method enhances the clinical relevance of the study. However, several notable limitations must be acknowledged. The retrospective design of the study may have introduced selection and recall biases. The small sample size of 84 participants may limit the generalizability of the findings and hinder the feasibility of conducting subgroup analyses. The single-facility setting may also constrain the applicability of these results to other clinical environments. Another limitation is the variability in the timing of maternal blood sampling and the lack of longitudinal assessment of corisin level dynamics throughout pregnancy. Another limitation of this study is the lack of stratification by small for gestational age/intrauterine growth restriction versus preterm appropriate for gestational age infants due to the limited number of LBW cases. Addressing these limitations in future prospective studies with larger populations will be essential to validate the potential role of corisin in adverse pregnancy outcomes.

## 5. Conclusions

In conclusion, this exploratory study found that pregnant women who experienced preterm birth or delivered low-birth-weight infants exhibited significantly altered serum levels of corisin compared with controls. Corisin levels were also significantly correlated with several perinatal outcomes, markers of coagulation activation, and a marker of oxidative stress. These associations raise the possibility that corisin may be linked to adverse pregnancy outcomes. However, given the observational nature of the study and the limited sample size, well-powered prospective investigations are needed to confirm these findings and better understand the underlying mechanisms. Such studies may ultimately help assess whether corisin has utility as a predictive or mechanistic biomarker in maternal-fetal medicine.

## 6. Patents

E.C.G., C.N.D.-G., and I.C. hold a patent on corisin and the anticorisin monoclonal antibody reported in this study.

## Figures and Tables

**Table 1:** Prenatal and perinatal clinical data of patients with term and preterm birth.

Variable	Term Birth	Preterm Birth	*p* Values	All Subjects
No of subjects	74	10		84
* Underlying diseases			0.153	
‡ None	47 (63.5%)	9 (90.0%)		56 (66.7%)
With underlying diseases	27 (37.8%)	1 (10.0%)		28 (33.3%)
Type 2 diabetes mellitus	2 (2.7%)	0		2 (2.4%)
Arterial hypertension	5 (6.8%)	0		5 (5.9%)
Hypothyroidism	2 (2.7%)	0		2 (2.4%)
Pulmonary sarcoidosis	1 (1.4%)	0		1 (1.2%)
Hereditary Spherocytosis	1 (1.4%)	0		1 (1.2%)
Congenital antithrombin deficiency	1 (1.4%)	0		1 (1.2%)
Bronchial asthma	1 (1.4%)	0		1 (1.2%)
Familial hypercholesterolemia	1 (1.4%)	0		1 (1.2%)
Hashimoto’s disease	1 (1.4%)	0		1 (1.2%)
Graves’ disease	0	1 (10%)		1 (1.2%)
IgA nephropathy	1 (1.2%)	0		1 (1.1%)
Antiphospholipid syndrome	2 (2.7%)	0		2 (2.4%)
SS-A antibody positive	1 (1.4%)	0		1 (1.2%)
Psychiatric disorder				
Panic disorder	2 (2.7%)	1 (10%)		3 (3.6%)
Depression	2 (2.7%)	0		2 (2.4%)
Bipolar disorder	1 (1.4%)	0		1 (1.2%)
Anxiety disorder	1 (1.4%)	0		1 (1.2%)
Tumor				
Tongue cancer	1 (1.4%)	0		1 (1.2%)
Uterine fibroids/adenomyosis	1 (1.4%)	0		1 (1.2%)
§ Complications			0.091	
None	46 (62.2%)	3 (30%)		49 (58.3%)
With complications	28 (37.8%)	7 (70%)		35 (41.7%)
Preeclampsia	3 (4.1%)	1 (10%)		4 (4.8%)
Gestational diabetes mellitus	7 (9.5%)	0		7 (8.3%)
Hypertensive disorder of pregnancy	2 (2.7%)	1 (10%)		3 (3.6%)
Preterm labor	-	3 (30%)		3 (3.6%)
Chorioamnionitis/chorangiosis	14 (18.9%)	4 (40%)		18 (21.4%)
Fetal growth restriction	4 (5.4%)	0 (0%)		4 (4.8%)
Non-reassuring fetal status	7 (9.5%)	2 (20%)		9 (10.7%)
Small-for-gestational-age infants	7 (9.5)	1 (10%)	0.999	8 (9.5%)
Methods of childbirth			0.374	
Cesarean section	62 (83.8%)	7 (70%)		69 (82.1%)
Natural delivery	12 (16.2%)	3 (30%)		15 (17.9%)
Methods of conceptions			0.273	
Natural	47 (63.5%)	9 (90%)		56 (66.7%)
Hormone replacement cycle frozen- thawed embryo transfer.	24 (32.4%)	1 (10%)		25 (29.8%)
Natural cycle frozen transfer	3 (4.1%)	0		3 (3.6%)

* Some cases have more than one underlying disease. ‡ Patients with complications are included. § Some patients have more than one complication.

**Table 2:** Prenatal and perinatal clinical data of patients by newborn birth weight group.

Variable	Newborn Body Weight > 2.5 kg	Newborn Body Weight < 2.5 kg	*p* Values	All Subjects
No of subjects	62	22		84
* Underlying diseases			0.114	
‡ None	38 (61.3%)	18 (81.8%)		56 (66.7%)
With underlying diseases	24 (38.7%)	4 (18.2%)		28 (33.3%)
Type 2 diabetes mellitus	2 (3.2%)	0		2 (2.4%)
Arterial hypertension	5 (8.1%)	0		5 (5.9%)
Hypothyroidism	2 (3.2%)	1 (5.0%)		3 (3.6%)
Pulmonary sarcoidosis	1 (1.6%)	0		1 (1.2%)
Hereditary Spherocytosis	1 (1.6%)	0		1 (1.2%)
Congenital antithrombin deficiency	1 (1.6%)	0		1 (1.2%)
Bronchial asthma	1 (1.6%)	1 (5.0%)		2 (2.4%)
Familial hypercholesterolemia	1 (1.6%)	0		1 (1.2%)
Hashimoto’s disease	1 (1.6%)	0		1 (1.2%)
Graves’ disease	0	1 (5.0%)		1 (1.2%)
IgA nephropathy	1 (1.6%)	0		1 (1.2%)
Antiphospholipid syndrome	2 (3.2%)	0		2 (2.4%)
SS-A antibody positive	1 (1.6%)	0		1 (1.2%)
Others	2 (3.2%)	0		2 (2.4%)
Psychiatric disorder				
Panic disorder	1 (1.6%)	2 (9.1%)		3 (3.6%)
Depression	2 (3.2%)	0		2 (2.4%)
Bipolar disorder	0	1 (5.0%)		1 (1.2%)
Anxiety disorder	1 (1.6%)	0		1 (1.2%)
Tumor				
Tongue cancer	1 (1.6%)	0		1 (1.2%)
Uterine fibroids/adenomyosis	1 (1.6%)	0		1 (1.2%)
§ Complications				
None	44 (71.0.%)	5 (22.7%)	0.0001	49 (58.3%)
With complications	18 (29.0%)	17 (77.3%)		35 (41.7%)
Preeclampsia	0	4 (18.2%)		4 (4.8%)
Gestational diabetes mellitus	7 (11.3%)	0		7 (8.3%)
Hypertensive disorder of pregnancy	2 (3.2%)	1 (4.5%)		3 (3.6%)
Preterm labor	0 (0%)	3 (13.6%)		3 (3.6%)
Chorioamnionitis/chorangiosis	10 (16.1%)	8 (36.4%)		18 (21.4%)
Fetal growth restriction	0 (%)	4 (18.2%)		4 (4.8%)
Non-reassuring fetal status	3 (4.8%)	6 (27.3%)		9 (10.7%)
Small-for-gestational-age infants	1 (1.6%)	7 (31.8%)	0.0002	8 (9.5%)
Methods of childbirth			0.749	
Cesarean section	50 (80.7%)	19 (86.4%)		69 (82.1%)
Natural delivery	12 (19.4%)	3 (13.6%)		15 (17.9%)
Methods of conceptions			0.281	
Natural	38 (61.3%)	18 (81.8%)		56 (66.7%)
Hormone replacement cycle frozen- thawed embryo transfer.	20 (32.3%)	4 (18.2%)		24 (28.6%)
Natural cycle frozen transfer	3 (4.8%)	0		3 (3.6%)

* Some cases have more than one underlying disease. ‡ Patients with complications are included. § Some patients have more than one complication.

## Data Availability

All data generated or analyzed during the current study are included in the article. Also, any data and materials are available from the corresponding authors upon reasonable request.

## Supplementary Materials

The following supporting information can be downloaded at: https://www.mdpi.com/article/10.3390/antiox14060670/s1. Figure S1. Eligible patients. A total of 105 patients were retrospectively enrolled. Figure S2. Circulating corisin levels in patients with or without pregnancy-related complications and/or comorbidities. Serum corisin levels were measured by enzyme immunoassay, as described in the Materials and Methods section, in women with both pregnancy-related complications and comorbidities (*n* = 9), complications alone (*n* = 26), comorbidities alone (*n* = 19), and in those without either condition (*n* = 30). Data are presented as mean ± SEM. Statistical analysis was performed using an unpaired *t*-test. However, only 84 met the eligibility criteria for inclusion in the study. Figure S3. Correlation among various variables in all pregnant women. Correlation analysis among several variables, including corisin, in pregnant women (*n* = 84). Figure S4. No significant differences in circulating levels of an intestinal epithelial marker or correlation with serum corisin. A, B, Serum levels of fatty acid binding protein 2 (FABP2), a marker of intestinal epithelial integrity, were measured using an enzyme-linked immunosorbent assay, as detailed in the Materials and Methods section. The sample sizes were as follows: term deliveries, *n* = 64; preterm deliveries, *n* = 10; normal birth weight infants, *n* = 62; and low birth weight infants, *n* = 22. Data are presented as medians with interquartile ranges. Statistical analysis was conducted using the Mann–Whitney U test. Table S1. Clinical and laboratory data of patients with term and preterm births. Table S2. Prenatal and perinatal data in newborns with body weights of less and more than 2.5 kg. Table S3. Univariate and multiple linear regression analysis with birth weight as the continuous dependent variable.
