# Supplementary material for: Exploratory Evaluation of Circulating Microbiota-Derived Corisin Levels in Women with Adverse Pregnancy Outcomes
Source: Antioxidants (Basel). 2025 May 31;14(6):670. doi: 10.3390/antiox14060670 (PMC12189293; doi:10.3390/antiox14060670)
Supplement: Supplementary file 1 [file antioxidants-14-00670-s001.zip › antioxidants-3632463-supplementary.pdf]

Supplementary Information

Exploratory Evaluation of Circulating Microbiota-Derived Corisin Levels in Women with Adverse Pregnancy Outcomes

Maya Kato, Masafumi Nii, Kuniaki Toriyabe, Yuya Tamaishi, Sho Takakura, Shoichi Magawa, Taro Yasuma, Corina N. D'Alessandro-Gabazza, Hajime Fujimoto, Masaaki Toda, Isaac Cann, Tetsu Kobayashi, Esteban Gabazza, Eiji Kondo, Tomoaki Ikeda.

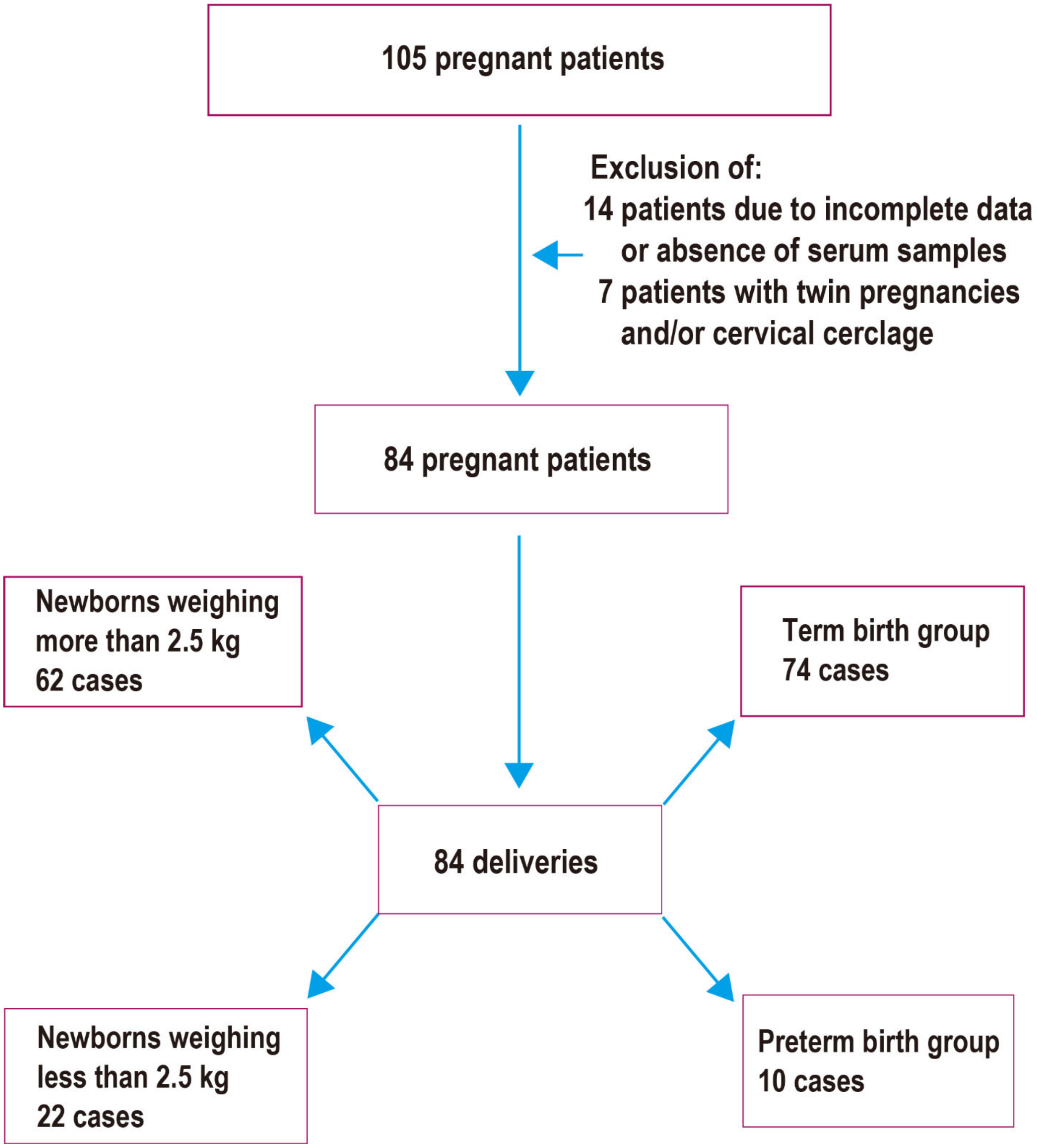

Supplementary Figure S1. Eligible patients. A total of 105 patients were retrospectively enrolled. However, only 84 met the eligibility criteria for inclusion in the study.

Supplementary Table S1. Clinical and laboratory data of patients with term and preterm births

	Term birth group	Preterm birth group	P values
**Mothers' data**			
No of subjects	74	10	
Age (years-old ± SEM)	34.8 ± 0.5	33.8 ±1.4	0.532
Body mass index	27.3 ± 0.6	24.6 ±1.6	0.142
Systolic pressure (mmHg)	116.2 ± 1.6	127.2 ± 7.4	0.038
Diastolic pressure (mmHg)	71.2 ± 1.8	76.5 ± 5.1	0.341
White blood cells (10^3/μL)	8459.5 ± 287.2	9131.0 ± 14.74.3	0.477
Blood platelets (104/μL)	22.8 ± 0.6	22.0 ± 1.9	0.637
Blood hemoglobin (g/dL)	11.2 ± 0.1	11.1 ± 0.2	0.878
Blood total protein (g/dL)	6.1 ± 0.0	6.1 ± 0.2	0.826
Blood albumin (g/dL)	2.9 ± 0.0	3.0 ± 0.1	0.949
Blood total bilirubin (mg/dL)	0.6 ± 0.0	0.6 ± 0.0	0.714
Serum aspartate aminotransferase (U/L)	17.8 ± 0.5	20.0 ± 1.3	0.193
Serum alanine transaminase (U/L)	10.7 ± 0.6	14.1 ± 2.0	0.062
Serum γglutamyl transpeptidase (U/L)	9.8 ± 0.8	10.6 ± 2.7	0.732
Glycemia (mg/dL)	83.5 ± 2.2	103.7 ± 11.0	0.008
Serum Na (mmol/L)	137.0 ± 0.2	136.7 ± 0.4	0.552
Serum K (mmol/L)	4.1 ± 0.0	3.8 ± 0.1	0.04
Serum Cl (mmol/L)	104.8 ± 0.2	104.6 ± 0.5	0.967
Serum C-reactive protein (mg/dL)	0.4 ± 0.0	0.5 ± 0.3	0.711
Blood urea nitrogen (mg/dL)	8.1 ± 0.2	7.9 ± 0.8	0.812
Creatinine (mg/dL)	0.5 ± 0.0	0.4 ± 0.0	0.518
Activated partial thromboplastin time (sec)	27.2 ± 0.2	27.7 ± 0.9	0.471
Prothrombin time (sec)	11.1 ± 0.0	10.9 ± 0.1	0.452
**Newborns' and placenta data**			
Estimated fetal weight (ultrasonography)(g)	2787.0 ± 51.3	2133.7 ± 135.9	0.0001
Placenta weight (g)	522.3 ± 13.7	441.8 ± 26.8	0.040
Height (cm)	48.2 ± 0.2	43.9 ± 0.5	<0.0001
Weight (g)	2885.8 ± 61.0	1997.8 ± 88.1	<0.0001
Head circumference (cm)	37.3 ± 3.9	30.9 ± 0.3	<0.0001
Apgar score (1 minute)	7.7 ± 0.1	6.7 ± 0.4	0.003
Apgar score (5 minutes)	8.7 ± 0.0	8.2 ± 0.2	0.008

Data are expressed as the mean ± standard error of the mean (SEM).

Supplementary Table S2. Prenatal and perinatal data in newborns with body weights of less and more than 2.5 kg

	Body weight >2.5 kg	Body weight <2.5 kg	P values
**Mothers' data**			
No of subjects	62	22	
Age (years-old ± SEM)	35.2 ± 0.6	33.1 ± 0.9	0.076
Body mass index	28.2 ± 0.7	23.8 ± 0.7	0.001
Systolic pressure (mmHg)	115.5 ± 1.7	123.1 ± 4.2	0.052
Diastolic pressure (mmHg)	71.0 ± 2.0	74.2 ± 3.6	0.424
White blood cells (103/μL)	8573.1 ± 318.4	8450.0 ± 752.6	0.859
Blood platelets (104/μL)	23.6 ± 0.6	20.3 ± 1.1	0.015
Blood hemoglobin (g/dL)	11.1 ± 0.1	11.2 ± 0.2	0.743
Blood total protein (g/dL)	6.2 ± 0.0	6.0 ± 0.1	0.164
Blood albumin (g/dL)	2.9 ± 0.0	2.9 ± 0.0	0.902
Blood total bilirubin (mg/dL)	0.6 ± 0.0	0.5 ± 0.0	0.409
Serum aspartate aminotransferase (U/L)	17.8 ± 0.6	18.8 ± 1.0	0.434
Serum alanine transaminase (U/L)	11.0 ± 0.7	11.3 ± 1.0	0.854
Serum γglutamyl transpeptidase (U/L)	9.8 ± 0.9	10.2 ± 1.5	0.812
Glycemia (mg/dL)	85.6 ± 2.4	86.5 ± 6.8	0.874
Serum Na (mmol/L)	136.8 ± 0.2	137.3 ± 0.3	0.303
Serum K (mmol/L)	4.0 ± 0.0	4.1 ± 0.1	0.952
Serum Cl (mmol/L)	104.7 ± 0.2	105.0 ± 0.3	0.613
Serum C-reactive protein (mg/dL)	0.5 ± 0.1	0.3 ± 0.1	0.304
Blood urea nitrogen (mg/dL)	7.9 ± 0.2	8.5 ± 0.5	0.318
Serum creatinine (mg/dL)	0.5 ± 0.0	0.5 ± 0.0	0.643
Activated partial thromboplastin time (sec)	27.0 ± 0.2	28.0 ± 0.4	0.04
Prothrombin time (sec)	11.2 ± 0.0	10.8 ± 0.1	0.03
**Newborns' and placenta data**			
Estimated fetal weight (ultrasonography)(g)	28.29.0 ± 42.2	2128.5 ± 66.8	<0.0001
Placenta weight (g)	553.7 ± 12.4	397.0 ± 17.8	<0.0001
Height (cm)	48.8 ± 0.2	44.3 ± 0.3	<0.0001
Weight (g)	3043.2 ± 51.7	2038.6 ± 51.8	<0.0001
Head circumference (cm)	38.4 ± 4.7	31.2 ± 0.2	<0.0001
Apgar score (1 minute)	7.8 ± 0.1	7.0 ± 0.2	0.002
Apgar score (5 minutes)	8.8 ± 0.0	8.3 ± 0.2	0.001

Data are expressed as the mean ± standard error of the mean (SEM).

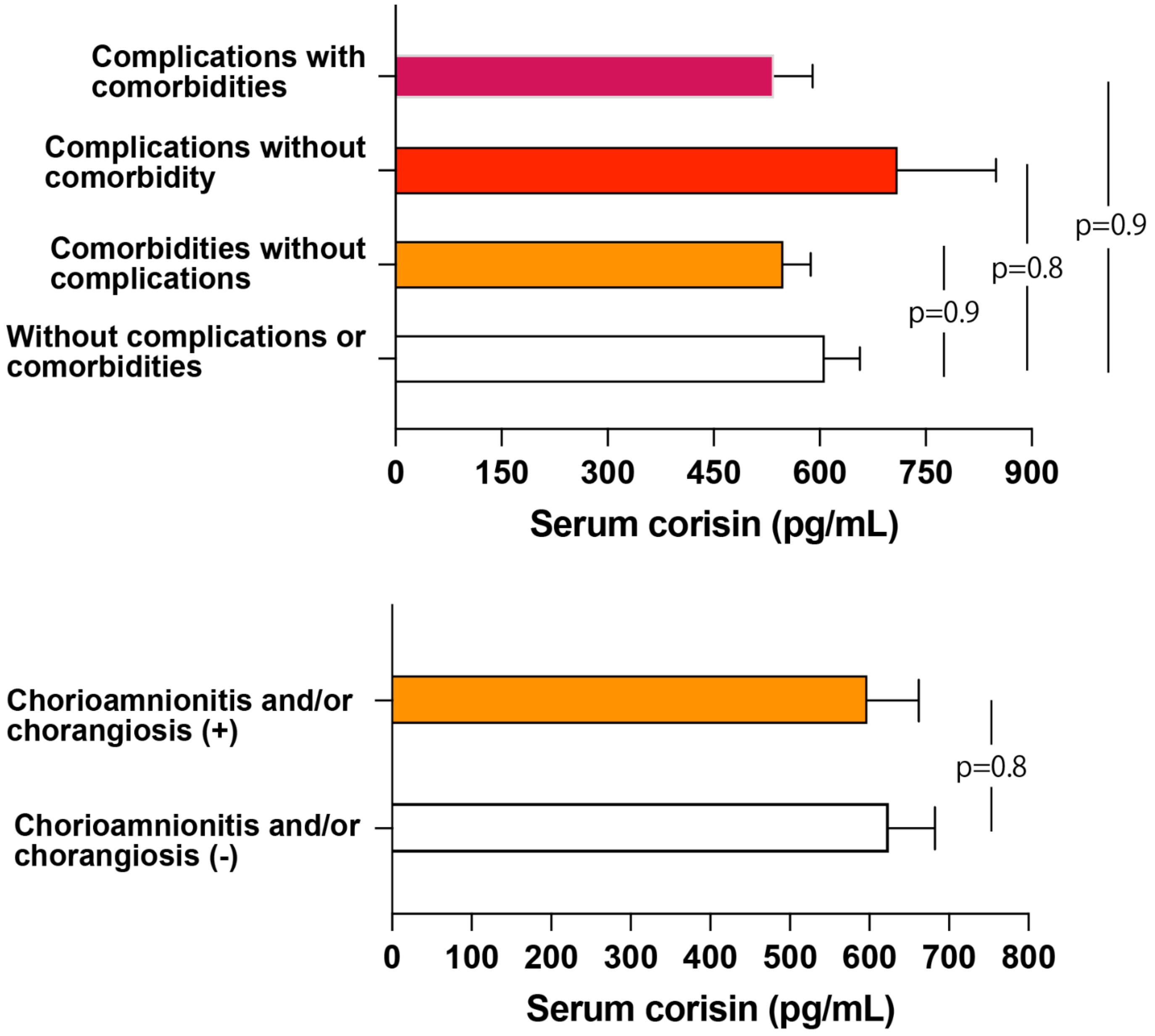

Supplementary Figure S2. Circulating corisin levels in patients with or without pregnancy-related complications and/or comorbidities. Serum corisin levels were measured by enzyme immunoassay, as described in the Materials and Methods section, in women with both pregnancy-related complications and comorbidities (n=9), complications alone (n=26), comorbidities alone (n = 19), and in those without either condition (n=30). Data are presented as mean ± SEM. Statistical analysis was performed using an unpaired t-test.

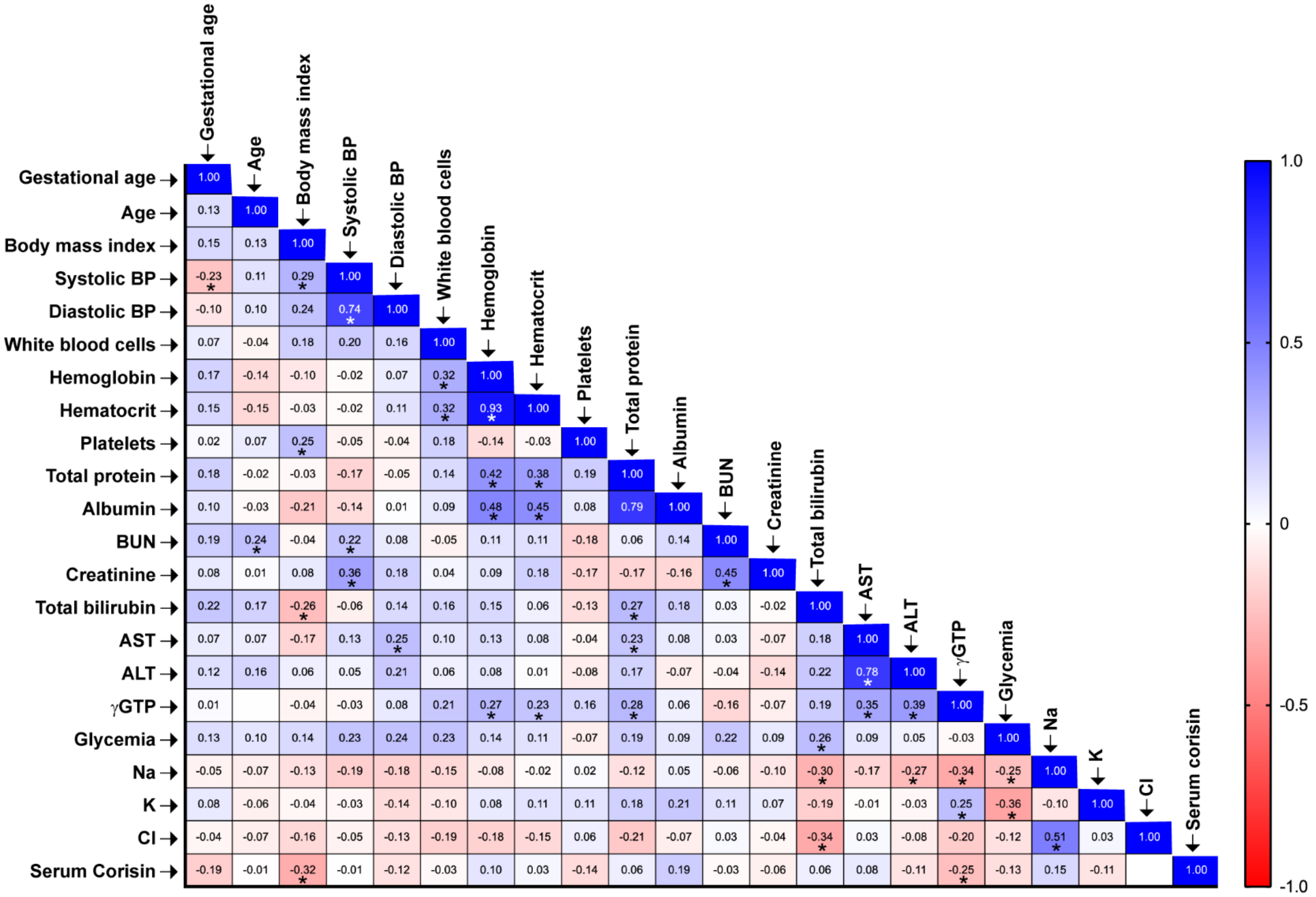

Supplementary Figure S3. Correlation among various variables in all pregnant women. Correlation analysis among several variables, including corisin, in pregnant women (n=84).

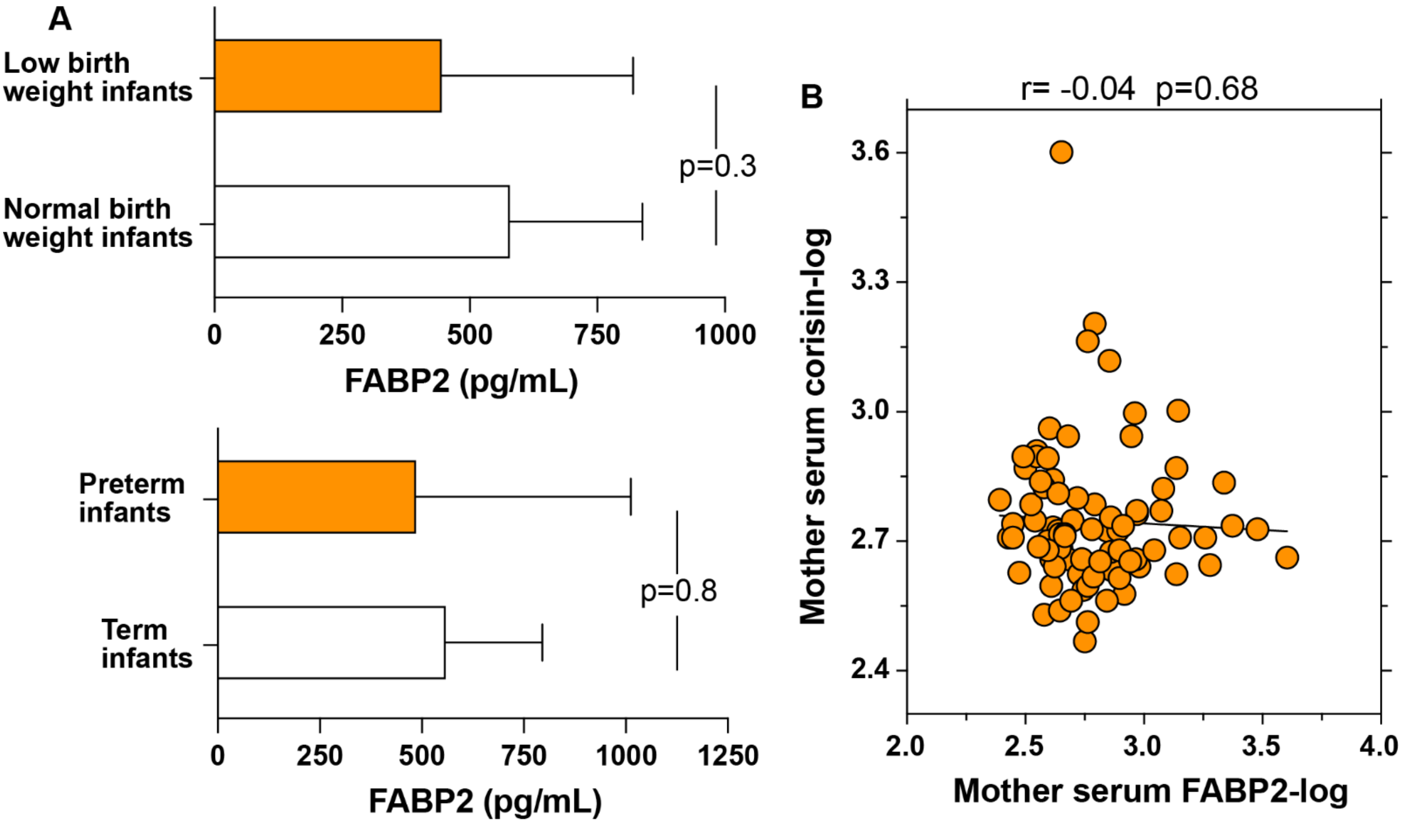

Supplementary Figure S4. No significant differences in circulating levels of an intestinal epithelial marker or correlation with serum corisin. A, B, Serum levels of fatty acid binding protein 2 (FABP2), a marker of intestinal epithelial integrity, were measured using an enzyme-linked immunosorbent assay, as detailed in the Materials and Methods section. The sample sizes were as follows: term deliveries, n = 64; preterm deliveries, n = 10; normal birth weight infants, n = 62; and low birth weight infants, n = 22. Data are presented as medians with interquartile ranges. Statistical analysis was conducted using the Mann–Whitney U test.

Supplementary Table S3. Univariate and multiple linear regression analysis with birth weight as the continuous dependent variable

Mothers' data	β values (CI)	P values
**Univariate analysis**		
Age	16.53 (-9.523 to 42.58)	0.2105
Systolic blood pressure	-7.628 (-15.37 to 0.1156)	0.0534
Diastolic blood pressure	-2.605 (-10.38 to 5.169)	0.5069
White blood cells	0.01165 (-0.03455 to 0.05784)	0.6173
Blood platelets	23.32 (0.6524 to 45.98)	0.0439
Blood hemoglobin	-5.106 (-113.8 to 103.5)	0.9257
Blood total protein	31.34 (-206.5 to 269.2)	0.7938
Blood albumin	-288.9 (-684.2 to 106.5)	0.1498
Blood total bilirubin	233.5 (-369.7 to 836.7)	0.4432
Serum aspartate aminotransferase	-13.95 (-40.91 to 13.00)	0.3060
Serum alanine transaminase	2.284 (-22.19 to 26.75)	0.8531
Blood glucose	-1.198 (-8.121 to 5.725)	0.7309
Serum Na	-50.22 (-127.2 to 26.79)	0.1980
Serum K	-46.32 (-364.6 to 272.0)	0.7728
Serum Cl	-30.19 (-101.3 to 40.95)	0.4007
Blood creatinine	-446.1 (-1832 to 940.3)	0.5237
Serum C-reactive protein	74.01 (-93.66 to 241.7)	0.3818
Serum tissue factor	-2.256 (-4.361 to -0.1518)	0.0359
Serum thrombin-antithrombin complex	-8.284 (-16.21 to -0.3605)	0.0407
Serum thrombomodulin	-0.1745 (-0.2954 to -0.05357)	0.0052
Serum fibrinogen	2.233 (0.4266 to 4.039)	0.0161
Serum myeloperoxidase	1.293 (-10.75 to 13.34)	0.8314
Serum corisin	-862.7 (-1600 to -125.2)	0.0224
**Multiple linear regression analysis**		
Model 1		
Blood platelets	19.72 (-2.768 to 42.21)	0.0848
Serum corisin	-772.1 (-1515 to -29.40)	0.0418
Model 2		
Serum thrombin-antithrombin complex	-131.7 (-496.8 to 233.5)	0.4752
Serum corisin	-785.7 (-1556 to -15.52)	0.0457
Model 3		
Serum thrombomodulin	-0.1634 (-0.2821 to -0.04465)	0.0076
Serum corisin	-778.6 (-1491 to -65.89)	0.0327
Model 4		
Serum tissue factor	-1305 (-3303 to 693.8)	0.1977
Serum corisin	-674.2 (-1464 to 115.2)	0.0931
Model 5		
Serum fibrinogen	1.827 (-0.02168 to 3.676)	0.0527
Serum corisin	-688.8 (-1509 to 131.2)	0.0984
